# Multimodality Imaging in Cardiac Metastasis of Cutaneous Melanoma: Case Report and Systematic Review

**DOI:** 10.3390/jcdd13020084

**Published:** 2026-02-09

**Authors:** Karina L. Lara-Sampayo, Juan Carlos Ibarrola-Peña, Miranda de la Pena-Tamez, Jose A. Salinas-Casanova, Rafael Garcia, Carlos Jerjes-Sanchez, Jose Gildardo Paredes-Vazquez, Erasmo de la Pena-Almaguer

**Affiliations:** 1 Hospital General de Zona y de Medicina Familiar Zona 2 IMSS Monterrey, Monterrey 64010, Nuevo Leon, Mexico; a00839705@tec.mx; 2 Escuela de Medicina y Ciencias de la Salud, Tecnológico de Monterrey, Monterrey 64710, Nuevo Leon, Mexico; jcibarrola.md@gmail.com (J.C.I.-P.); a01722919@tec.mx (M.d.l.P.-T.); alf.sc91@gmail.com (J.A.S.-C.); rafaelrodgr@gmail.com (R.G.); carlosjerjes@tec.mx (C.J.-S.); dr.jose.paredes.vazquez@gmail.com (J.G.P.-V.); 3 Instituto de Cardiologia y Medicina Vascular, TecSalud, Escuela de Medicina y Ciencias de la Salud, Tecnologico de Monterrey, San Pedro Garza Garcia 66260, Nuevo Leon, Mexico

**Keywords:** cardiac metastases, metastatic melanoma, cardiovascular magnetic resonance imaging, PET-CT

## Abstract

Background: Cardiac metastases from cutaneous melanoma are uncommon and often underdiagnosed due to their variable and frequently asymptomatic presentation. To better describe their clinical features, diagnostic strategies, and outcomes, we performed a systematic review of published case reports and present an illustrative clinical case. Case presentation: We report the case of a 67-year-old man with a history of stage IIA cutaneous melanoma who presented with progressive fatigue and dyspnea. Disease recurrence was confirmed by skin biopsy. Multimodal imaging, including echocardiography, FDG PET-CT, and cardiac magnetic resonance (CMR), demonstrated extensive myocardial infiltration consistent with cardiac metastases. Despite treatment with immunotherapy, the patient experienced progressive clinical deterioration and died six months after diagnosis. Discussion: The systematic review encompassed 23 published articles reporting 27 individual cases, with a mean age at diagnosis of 55.9 years and a clear male predominance. Cardiac involvement exhibited marked heterogeneity in both clinical presentation and anatomical distribution, most frequently affecting the left ventricular free wall and the interventricular septum. Echocardiography consistently served as the initial diagnostic modality, while cardiac magnetic resonance and CT/FDG PET-CT were used to refine lesion characterization and assess extracardiac disease. Notably, a complete multimodal imaging strategy was reported in fewer than one-third of cases, reflecting variability in diagnostic approaches. Survival outcomes were highly heterogeneous, with substantial mortality, underscoring the need for earlier detection and more accurate diagnostic strategies for cardiac involvement in melanoma. Conclusions: Cardiac metastases from melanoma represent advanced disease and remain associated with poor and heterogeneous outcomes. An integrated multimodal imaging approach supports detailed diagnostic characterization and may aid clinical evaluation and management in selected cases.

## 1. Introduction

Cardiac metastases from cutaneous melanoma are relatively common upon autopsy (50–65%) of advanced cases, but they are rarely diagnosed antemortem (<1–2%) [1]. Propensity for wide hematogenous dissemination, attributable to its embryological origin from neural crest cells, explains its ability to invade virtually any organ, including the heart [2]. This often-silent presentation can result in delayed recognition and limit opportunities for early intervention [3]. The most common signs and symptoms are nonspecific, including fatigue, tachycardia, dyspnea, arrhythmias, superior vena cava syndrome, transient ischemic attacks, congestive heart failure, pericardial effusion, edema, and right ventricular inflow or outflow obstruction [4].

Cardiac metastases traditionally signal a poor prognosis, reflecting advanced disease stage. The diagnostic approach to cardiac metastases requires the complementary use of multiple imaging modalities, each providing different but crucial information: echocardiography as an initial accessible tool, cardiovascular magnetic resonance imaging (CMR) for detailed tissue characterization, and a positron emission tomography-computed tomography (PET-CT) to assess the systemic extent of the disease and guide comprehensive treatment [5].

Early and systematic use of a multimodal imaging approach is crucial for the accurate detection of cardiac involvement and the management of these complex cases. We present a case that highlights the aggressive nature of cutaneous melanoma and emphasizes the importance of a comprehensive cardiac evaluation in patients with a known history of this malignancy. Additionally, we conducted a systematic review of the literature. This review aims to identify and analyze published case reports of cardiac metastases secondary to melanoma, with a specific focus on those originating from cutaneous melanoma. The literature search was performed using the PubMed database, applying the following combination of MeSH terms and free-text keywords: (Heart Neoplasms/secondary [MeSH] OR Cardiac metastasis [tiab]) AND (Melanoma [MeSH] OR cutaneous melanoma [tiab]) AND (Echocardiography [MeSH] OR Magnetic Resonance Imaging [MeSH] OR Positron-Emission Tomography [MeSH]) AND (Case Reports [Publication Type] OR case report [tiab]). This search strategy yielded 57 case reports. Each report was reviewed to confirm the cutaneous origin of the primary melanoma and to systematically extract relevant clinical, imaging, therapeutic, and outcome data. These data formed the basis for a comprehensive case-based review and a comparative analysis of the clinical and imaging patterns associated with cardiac metastases in cutaneous melanoma.

## 2. Case Presentation

A 67-year-old man presented with a three-month history of progressive fatigue and dyspnea. Grayish pigmented lesions were noted on the trunk and perioral region, the largest measuring approximately 1 cm. Past medical history was significant for stage IIA cutaneous melanoma, diagnosed in 2018 and managed with surgical excision without lymphatic involvement. However, follow-up was discontinued in 2020 due to the COVID-19 pandemic. On physical examination, no additional abnormalities were detected.

**Figure 1:**
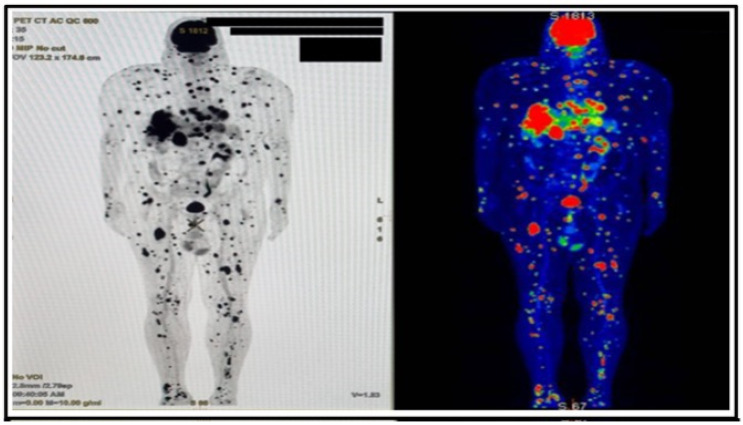
PET/CT demonstrates multiple hypermetabolic lesions, primarily localized in the liver, pancreas, and lungs, with additional findings suggestive of cardiac hypermetabolic involvement.

A skin biopsy from one of the suspicious lesions confirmed recurrent malignant melanoma, with molecular analysis revealing a positive BRAF mutation and PD-L1 expression. Given the symptoms, concern for cardiac metastatic spread prompted further evaluation. An electrocardiogram (ECG) showed no repolarization abnormalities. Transthoracic echocardiography showed mild left atrial enlargement and inferolateral left ventricular hypertrophy, with a mildly heterogeneous infiltrative appearance, which, in the clinical context, was suggestive of an infiltrative process. Biventricular systolic function and regional wall motion were preserved, with mild left ventricular diastolic dysfunction. The DEM score was 3 points, considered suggestive of malignancy; however, no discrete intracardiac mass was identified, and no significant valvular abnormalities were observed [6]. Considering the history of melanoma and the new onset of fatigue and dyspnea, a PET-CT was performed (Figure 1), which demonstrated multiple hypermetabolic lesions in the liver, pancreas, lungs, bones, and subcutaneous/mesenteric fat, as well as suspicious uptake within the heart.

**Figure 2:**
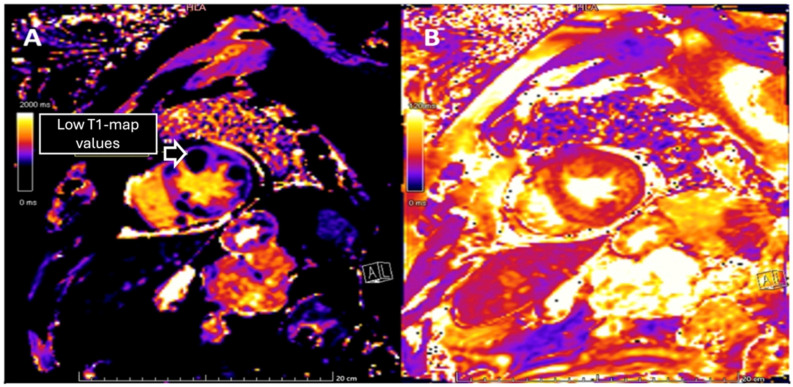
(**A**) Short-axis image from a T1 parametric map revealing multiple focal areas with decreased T1 signal, illustrating zones of necrosis. (**B**) Short-axis image from T2 parametric map demonstrating heterogeneous perilesional edema, suggestive of lesion-associated inflammation.

**Figure 3:**
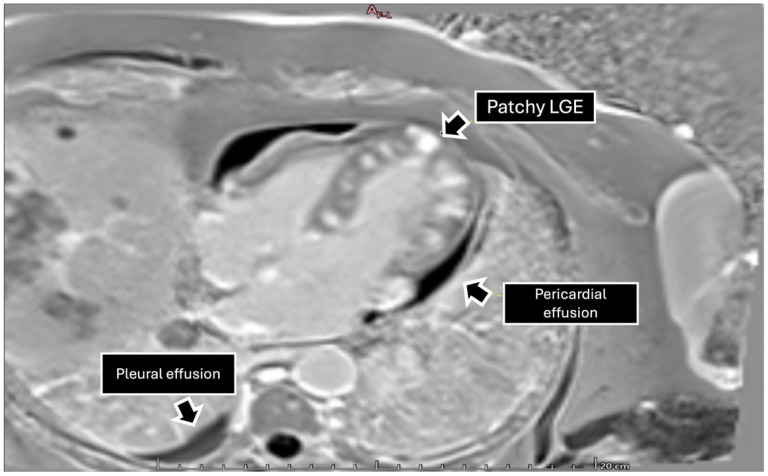
Four-chamber view with late gadolinium enhancement sequence showing areas of diffuse enhancement in the left ventricular wall, especially in the atrioventricular groove corresponding to the right coronary artery territory. Pericardial thickening with a mild pericardial effusion is observed. Extended images toward the lung reveal multiple diffusely distributed tumors, as well as a small right pleural effusion.

**Figure 4:**
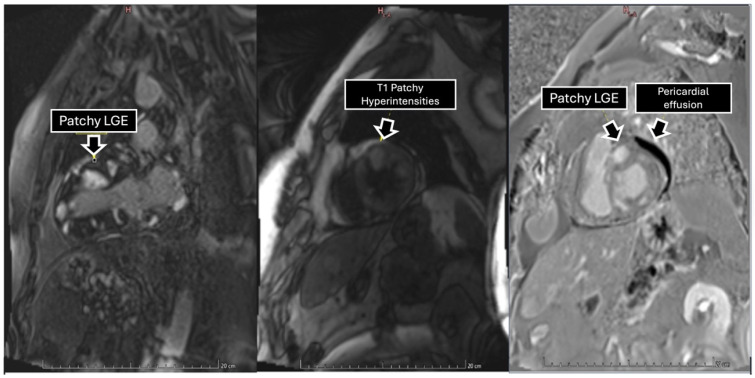
Late gadolinium enhancement sequence in two-chamber and short axis views depicting tumors with heterogeneous gadolinium uptake, reflecting extensive areas of intratumoral necrosis within the myocardial wall.

To further characterize the cardiac findings, we performed CMR, which demonstrated concentric left ventricular (LV) hypertrophy and normal global wall motion, with a preserved ejection fraction ≥70%. On T1-weighted sequences, heterogeneous cylindrical hyperintense regions with diffuse, ill-defined margins were observed throughout the LV myocardium, suggesting extensive infiltrative disease (Figure 2). Native T1 mapping revealed focal areas of low signal intensity, consistent with possible necrosis (Figure 3). Additionally, late gadolinium enhancement (LGE) demonstrated focal and diffuse predominantly mid-wall myocardial involvement, with heterogeneous gadolinium uptake in two-chamber and short-axis views, consistent with intramural necrosis and a non-coronary pattern, supporting metastatic infiltration rather than ischemic injury (Figure 4). It should be noted, however, that low native T1 values on CMR are not specific to cardiac melanoma metastases and may also be observed in other myocardial storage diseases, including myocardial iron/hemosiderin deposition and Anderson–Fabry disease [7]. Nevertheless, in the appropriate clinical context, a structured CMR-based assessment may provide incremental diagnostic value. In our patient, the CMR MASS score was 6 points, driven by infiltrative myocardial involvement, a polylobulated mass-like morphology, associated pericardial effusion, and early contrast enhancement. When low native T1 values were integrated with the patient’s oncologic history and systemic disease burden, these findings were consistent with malignant cardiac involvement.

**Figure 5:**
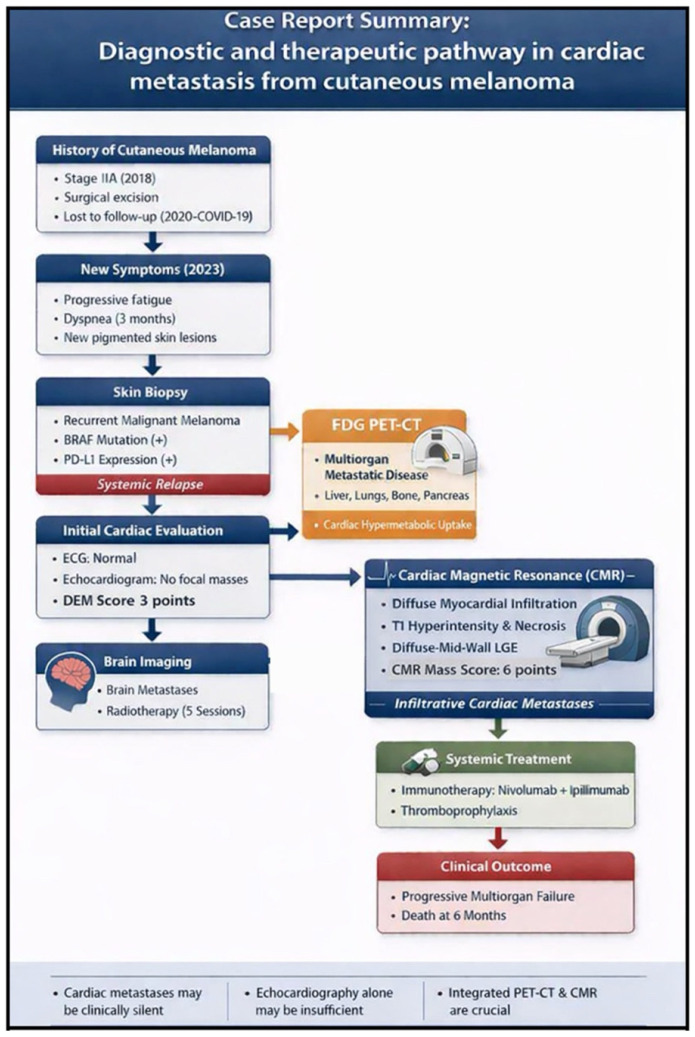
Case Report Summary: Diagnostic and Therapeutic Pathway.

Subsequent imaging identified brain metastases, for which the patient received five sessions of radiotherapy to the head and neck. Combined immunotherapy with nivolumab and ipilimumab was initiated in accordance with standard treatment guidelines. Anticoagulant therapy was not routinely indicated, as the patient had no established indications such as atrial fibrillation or venous thromboembolism; however, given the presence of active malignancy and cardiac involvement, which confer an increased risk of cancer-associated hypercoagulability and thromboembolic events, a Khorana Risk Score for venous thromboembolism of 2 points (intermediate risk) was calculated, and thromboprophylaxis was administered during hospitalization [8]. The patient was followed closely by a multidisciplinary team, with monitoring of both systemic and cardiac disease progression. However, six months later, multiorgan failure developed, resulting in death. A schematic summary of the case is provided in Figure 5.

## 3. Discussion

**Figure 6:**
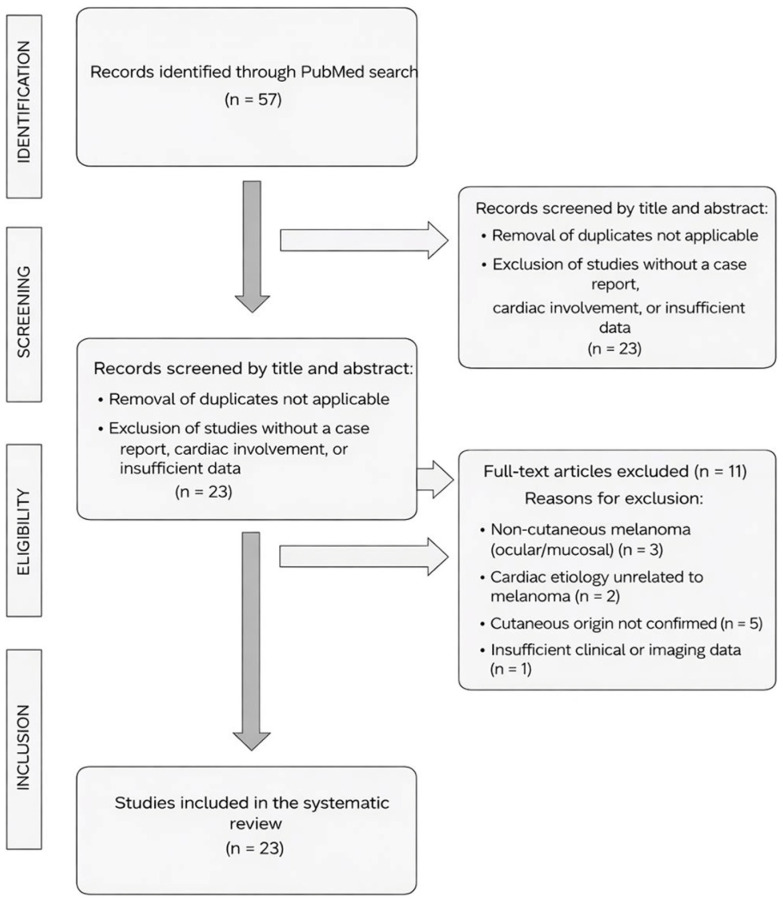
PRISMA Flow Diagram.

Cardiac metastases from cutaneous melanoma represent a rare but clinically significant manifestation of metastatic progression and are associated with substantial morbidity and mortality. This systematic review of the PubMed database, conducted by two independent reviewers with third-reviewer adjudication for discrepancies, included 27 individual cases from 23 published articles (Figure 6). Marked heterogeneity was observed in clinical presentation, timing of recurrence, anatomical distribution of cardiac involvement, and diagnostic approaches. The mean age at diagnosis was 55.9 years, consistent with previously reported series of advanced melanoma, and a male predominance was observed, with approximately two-thirds of cases occurring in men, likely reflecting both biological factors associated with more aggressive disease and behavioral factors such as lower risk awareness, delayed self-detection, and later presentation to medical care in males [9]. A detailed summary of the individual cases is provided in Table 1.

This review included only cases of cutaneous melanoma, excluding non-cutaneous melanomas due to their distinct clinical, biological, and prognostic characteristics [33]. Likewise, due to the methodology used, studies in which it was not possible to determine the classification of melanoma were excluded. Mucosal melanomas, which account for approximately 1% of all melanomas, are typically diagnosed at advanced stages due to their hidden anatomical locations and are not associated with ultraviolet radiation exposure. Furthermore, they exhibit more aggressive behavior, have a poorer prognosis, and are less responsive to standard immunotherapy, necessitating different therapeutic strategies [34,35]. The lack of randomized clinical trials and specific treatment guidelines for these subtypes reinforces the need to analyze them separately.

Echocardiography remains the first-line imaging technique for suspected cardiac masses due to its accessibility, portability, and low cost [36]. It provides valuable information on intracavitary or intramyocardial lesions, pericardial effusions, valvular abnormalities, and indirect signs such as papillary muscle infiltration. However, echocardiographic findings can sometimes be nonspecific, and image quality is operator dependent. In the reviewed cases, more than half showed echocardiographic signs suggestive of masses or tumors, underscoring its role as an initial diagnostic tool. Nonetheless, as noted previously, its findings are often nonspecific and may not confirm metastatic involvement, as demonstrated in our case.

Detecting cardiac metastases requires a robust diagnostic framework due to their variable presentation and frequently asymptomatic course. The implementation of a multimodal imaging strategy that integrates complementary techniques substantially enhances lesion detection and characterization. This approach improves diagnostic accuracy and facilitates more informed treatment planning and timing. The combined use of echocardiography, CT, CMR, and functional imaging enables a more comprehensive assessment of cardiac involvement and extracardiac disease, thereby supporting timely and appropriate therapeutic decision-making. However, among cases evaluated using a multimodal diagnostic approach, survival outcomes remained highly heterogeneous, with both long-term survivors and early mortality reported, precluding definitive conclusions regarding its impact on prognosis.

Cardiac metastases can involve the myocardium thickness, although the epicardium is the most common location. The pericardium and myocardium are commonly involved with multifocal, small lesions [37]. Within the myocardium, the free wall of the left ventricle and the interventricular septum are the most frequently affected regions, a pattern consistent with our clinical case. This predilection is likely related to their greater myocardial mass, rich coronary blood supply, and high perfusion, which facilitate hematogenous tumor cell seeding and infiltration [4]. In contrast, the endocardium is less frequently involved; when affected, it typically results from tumor extension into the cardiac chambers and may produce intracavitary lesions capable of obstructing ventricular outflow. According to current clinical standards, CMR is considered the gold standard for tissue characterization, due to its excellent spatial resolution and versatile pulse sequences. One significant advantage of CMR over other imaging techniques, such as CT, is that it does not involve ionizing radiation, making it a safer option for patients. Additionally, its excellent contrast resolution allows for a more precise assessment of the nature and origin of cardiac and pericardial masses. CMR also excels in providing high-resolution anatomical detail, often clarifying complex anatomical relationships. This detailed imaging capability enables the accurate determination of tumor extent, facilitating more effective surgical or ablative planning [13].

In cutaneous melanoma cases, lesions are commonly hyperintense on T1-weighted images due to melanin’s paramagnetic effects, although amelanotic variants may not follow this pattern [38]. The use of LGE sequences is especially valuable for detecting diffuse infiltration that may not correspond to a coronary artery territory [39]. In our review, over half of the cases included CMR, facilitating improved diagnostic planning and therapeutic decisions. In our patient, CMR provided detailed, personalized clinical insights. These findings highlight the importance of integrating CMR into diagnostic workflows, as it allows for anatomical and tissue characterization that other modalities may overlook.

When there is suspicion of metastatic spread, it becomes crucial to use additional imaging techniques that provide a more comprehensive and detailed assessment. CT is commonly used for both initial staging and follow-up, mainly due to its widespread availability, cost-effectiveness, and reasonable accuracy. In patients with cutaneous melanoma, CT has an overall sensitivity of approximately 58% and a specificity of nearly 70% for identifying metastases. PET-CT has substantially improved diagnostic capabilities in metastatic melanoma [40]. This advanced imaging modality is valuable not only for diagnosis but also for staging and ongoing monitoring. By providing whole-body imaging in a single session, PET-CT enables the detection of metastases throughout the body, including the heart and other organs. A key advantage is its ability to detect metabolic changes before structural abnormalities become evident [41]. Specifically, FDG-PET-CT exhibits superior sensitivity (~98.7%) compared to FDG-PET alone (~88.9%) and CT alone (~69.7%) [42]. Since melanoma metastases avidly uptake the FDG tracer, standardized uptake values (SUVs) assist in differentiating normal cardiac tissue from metastatic lesions, improving diagnostic confidence and guiding treatment planning. Although fewer than 25% of patients in our review underwent PET imaging, most of these demonstrated clinically relevant findings with diagnostic and prognostic value, supporting the expanding role of PET-CT in the evaluation of metastatic cutaneous melanoma.

From a diagnostic standpoint, echocardiography was the most frequently reported imaging modality, used in approximately 80% of cases and typically employed as the first-line diagnostic tool. However, echocardiography alone was often insufficient for comprehensive lesion characterization. Cardiac magnetic resonance imaging emerged as the second most frequently utilized imaging modality, reflecting its key role in tissue characterization and assessment of myocardial and pericardial involvement. In addition, CT and/or FDG PET-CT were used in approximately half of the cases to further delineate tumor extent and evaluate extracardiac disease burden. Notably, nearly two-thirds of patients required a multimodal imaging approach, while only a minority of cases (approximately one-third) had all three imaging modalities—echocardiography, cardiac MRI, and CT/FDG PET-CT—reported.

Multimodality imaging is central to the evaluation of cardiac tumors, while endomyocardial biopsy is reserved for selected cases with inconclusive noninvasive findings. Endomyocardial biopsy is avoided in high-embolization-risk intracardiac masses, but when indicated, image guidance improves its safety and diagnostic yield [43,44,45].

Effective management of metastatic cardiac melanoma necessitates systemic therapy. While surgical resection may alleviate symptoms in isolated cardiac metastases, most patients present with disseminated disease, which is unsuitable for surgery. Conventional chemotherapy shows limited benefit; however, targeted therapies and immune checkpoint inhibitors (e.g., nivolumab and ipilimumab) have improved survival in metastatic melanoma, including cardiac involvement [39]. Available evidence suggests that immunotherapy and chemotherapy demonstrate comparable effectiveness in cardiac metastases to that observed in metastases at other organ sites, with immunotherapy showing concordant response rates and acceptable safety profiles in most patients. Overall survival is influenced by multiple factors, including the stage and biology of the primary malignancy, the presence of additional metastatic sites, the specific site of cardiac involvement, and the efficacy of supportive and palliative measures [46]. The use of palliative radiotherapy in cardiac metastases has been limited due to technical challenges and concerns regarding radiation-induced cardiac toxicity. However, it may be considered in selected cases for symptom control [47]. Early detection, genomic profiling to guide therapeutic prioritization, and personalized multimodal treatment strategies remain critical to optimizing outcomes. Ongoing research into novel immunotherapies holds promise for future advances.

Survival outcomes across the reported cases were highly heterogeneous, likely reflecting both the aggressive biological behavior of metastatic melanoma and the variability inherent to case-based reports. Among the 27 cases included, 11 patients were reported to have died, 6 were alive at last follow-up, and survival status was not reported in 10 cases, highlighting the substantial degree of incomplete outcome reporting in the literature. To reduce the potential for outcome overestimation and misclassification bias, only cases with explicitly reported vital status were included in survival assessments. Within this subset (n = 17), the mortality rate was 62.9%, underscoring the generally poor prognosis associated with cardiac involvement by melanoma. Most reported deaths occurred within a short period following diagnosis, often within weeks to months, whereas longer survival was observed primarily in selected patients who underwent surgical resection combined with systemic therapy.

**Figure 7:**
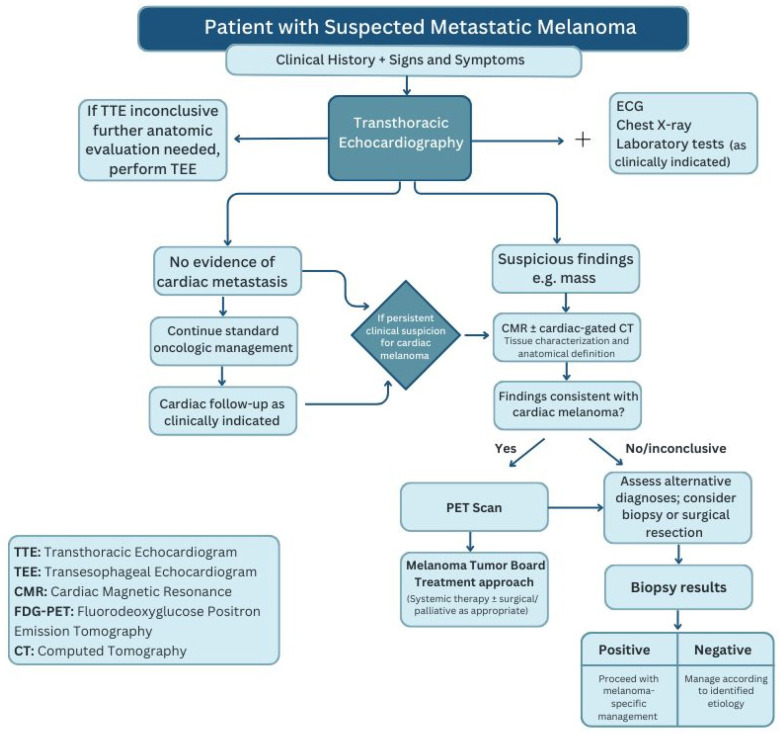
Multimodal diagnostic approach for suspected cardiac metastatic melanoma.

Our systematic review revealed a frequent lack of coordinated imaging protocols in reported cases, in contrast to the comprehensive workup conducted in our patient. Our evaluation included a focused physical exam for cutaneous recurrence and active screening for cardiovascular symptoms. ECG showed no repolarization abnormalities, followed by transthoracic echocardiography, PET/CT, and CMR, collectively providing precise delineation of disease extent. This structured, stepwise approach, summarized in Figure 7, contrasts with many reports in which some imaging modalities are omitted.

Although intracardiac masses are often documented with echocardiography, CT, or CMR, few studies employ an integrated imaging strategy that enables a comprehensive diagnostic evaluation. Additionally, key clinical variables—such as ECG findings, the interval between primary melanoma and cardiac metastasis, cardiovascular symptoms, treatment approaches, and patient outcomes—are inconsistently reported, limiting meaningful clinical interpretation. In our review, fewer than 25% of cases described multimodal imaging, including echocardiography, CMR, and CT/PET integration. Even in these, clinical progression and outcomes were variably documented, with most cases reporting fatal outcomes at publication. These observations highlight the importance of standardized, detailed reporting in future studies to improve diagnostic accuracy and inform clinical decision-making.

Interpretation of outcome data is inherently constrained by limitations intrinsic to the available literature, including retrospective, case-based reporting, short and heterogeneous follow-up durations, and incomplete documentation of clinical course and survival outcomes. These limitations restrict the ability to draw definitive conclusions regarding prognosis and necessitate cautious interpretation of the findings. Future studies would benefit from more standardized and comprehensive reporting of diagnostic pathways, imaging strategies, follow-up duration, and survival outcomes to improve the quality and comparability of evidence related to cardiac involvement in melanoma. In this context, the present case illustrates how a structured multimodal diagnostic approach can support detailed characterization of cardiac involvement and provide a comprehensive descriptive framework to inform clinical assessment in similar cases.

## 4. New Challenges Arise

Diagnosing intracavitary cardiac tumors remains a clinical challenge, particularly in melanoma patients, where cardiac metastases are both rare and often clinically silent. This reality demands a high index of suspicion, even in the absence of overt cardiovascular symptoms. Early implementation of integrated imaging techniques—combining structural, functional, and metabolic assessments—is crucial not only for accurate diagnosis but also for timely therapeutic planning. As systemic therapies evolve, especially with the emergence of immune checkpoint inhibitors, the landscape of treatment for metastatic melanoma continues to shift. Nevertheless, the prognosis for patients with cardiac involvement remains poor, highlighting the need for earlier detection strategies and robust follow-up protocols for high-risk populations [48].

## 5. Considerations

This case illustrates the aggressive nature of metastatic melanoma and emphasizes the necessity of detailed cardiovascular evaluation in patients with this malignancy. Utilizing an integrated imaging approach—particularly with CMR and PET-CT—enables precise lesion characterization and staging, both essential for informed clinical decisions. While CMR excels in tissue differentiation, PET-CT provides vital insights into systemic disease burden.

Despite advances in immunotherapy improving outcomes for some patients, many still present with advanced disease, limiting curative options. Early detection and multidisciplinary, individualized management are crucial for improving prognosis in this challenging population. Future efforts should focus on risk-adapted surveillance and the integration of emerging imaging biomarkers to enable earlier diagnosis and intervention.

## Figures and Tables

**Table 1:** Characteristics of the studies included in the systematic review.

Author	Time to Metastasis	Cardiovascular Symptoms	ECG	Echocardiography	CT/PET	RMI	Treatment Given	Clinical Outcome
**Haralabos Parissis et al. [10]**	7 months	NR	NR	Mildly dilated RA with a 5 × 3 cm mass occupying most of the atrial cavity	Focus of intense increased FDG uptake of activity in the RA and a 2.6 cm node in the left axilla	A 29 × 7 × 40 mm mass within the RA along the right postero-lateral wall, between the ostia for the superior and IVC	Surgery + ECMO + Vinblastine and Dacarbazine	Deceased
**François Roubille et al. [11]**	15 years	Dyspnea, worsening cough, strong systolic–diastolic murmur, and right cardiac failure signs	Sinus tachycardia	An intracardiac mass occupying at least three-quarters of the RV, extending into the outflow tract. A small portion of the tumor prolapses into the RA, while another extends through the interventricular septum into the LV. Circumferential pericardial effusion present	A tumor was identified with no evidence of recurrence	Heterogeneous enhancement of a tumor invading the interventricular septum, with a small extension into the LV	Chemotherapy + corticosteroids + dacarbazine.	The patient died three months after initiating treatment.
**Adriana Villa et al.** **[12]**	Unspecified years	Shortness of breath and fainting without chest pain	Atrial flutter 2:1	An irregular mass occupying the RA near the SVC	Cardiac masses exhibited polymorphic features, presenting both infiltrative and vegetative characteristics, with metastases detected in the brain, liver, and adrenal glands.	A polypoid atrial mass was located on the posterior wall of the RA, near the SVC. The intramyocardial and right atrial masses demonstrated different signal intensities, although most appeared hyperintense on both T1- and T2-weighted images. Additional masses were identified in the interatrial septum and RA, along with solid tissue infiltrating the ventricular myocardium bilaterally	Chemotherapy	Died around 1 year after the diagnosis.
**Elie Mousseaux et al. [13]**	4 years	NR	NR	Pericardial effusion accompanied by infiltration of the left ventricular wall	NR	Tumoral infiltrations from the lesion were identified involving both the pleura and pericardium. A large tumor measuring 50 mm was also observed along the interior walls of the LA and LV. The lesion showed enhancement following gadolinium administration	Carboplatin and interferon	The patient died two years after diagnosis
**Elie Mousseaux et al. [13]**	Unknown	NR	NR	Non-contributive	NR	Tumor within the lateral wall of the LV (30 × 26 mm) enhanced after gadolinium injection	Ablated	Recovered uneventfully and remained alive five years postoperatively
**Elie Mousseaux et al. [13]**	Unknown	NR	NR	Mild pericardial effusion, and left ventricular hypertrophy with two intramural masses, one in the septum and one at the apex.	NR	Large pericardial effusion with a large multifocal tumor involving the lateral wall of the LA and the atrial septum, obstructing the right ventricular outflow tract. The mass showed enhancement following gadolinium injection	Fotemustine	The patient died eight months after diagnosis
**Elie Mousseaux et al. [13]**	Unknown	Superior vena cava syndrome	Not reported	An 8-cm mass occupying the lumen of the RA	NR	The right atrial wall facing the implantation of the tumor was infiltrated into the adjacent pericardium as well. The superior vena cava was also heavily infiltrated	Surgical resection	The patient was alive one year post-surgery, with no evidence of additional metastases
**Faruk Tas et al. [14]**	6 months	Asymptomatic	Normal	A lesion measuring 0.3 cm in diameter was identified	Intense FDG uptake was observed in the interatrial region of the heart, accompanied by mediastinal lymphadenopathy and pulmonary parenchymal metastases, as well as the liver, bones, and soft tissues	NR	Temozolomide and Fotemustine	NR
**Mahmoud Houmsse et al. [15]**	4 years	Soft II/VI systolic ejection murmur along the lower left sternal border	Left axis deviation, left anterior fascicular block, and T wave inversion of leads V1–V3	A large mass occupying approximately three-quarters of the LV, likely originating from the apical region. The mass extended into the left ventricular outflow tract, reaching within 1 cm of the aortic valve annulus, and narrowed the outflow tract diameter to approximately 5 mm	A left ventricular mass	A mass located in the distal anteroseptal-apical region, characterized by high water content	Surgical excision of a 7 cm tumor via left posterolateral ventriculotomy+ chemotherapy + immunotherapy (two cycles of aldesleukin)	Asymptomatic
**C. J. Ellis et al. [16]**	6 years	Lethargy, fatigue, and night sweats.	Normal	Large pedunculated tumor of the left ventricle	Normal/NR	Infiltrative lesion	Local radiotherapy and single-agent chemotherapy	Died 10 weeks following the cardiac biopsy
**C. J. Ellis et al. [16]**	2 years	Dyspnea, marked peripheral swelling, large bilateral pleural effusions, and subsequently recurrent episodes of ventricular tachycardia, marked peripheral edema	Sinus rhythm with a right bundle branch block pattern	An adherent tumor mass in the right ventricle	Normal/NR	NR	Surgical debulking	Clinical recovery was favorable two months following cardiac surgery
**Monika J. Leja et al. [17]**	4 years	NR	NR	NR	A 2.5 cm intracavitary mass in the left ventricle and a 10 mm non-cardiac nodule in the left upper lung lobe	An isolated mass measuring 1.4 × 1.2 cm was located deep within the left ventricular cavity, attached to the inferolateral wall. The mass appeared isodense on both T1- and T2-weighted images, demonstrated hypoperfusion on first-pass imaging, and showed moderate delayed contrast enhancement	Systemic biochemotherapy with cisplatin, vinblastine, and temozolomide, followed sequentially by interferon and interleukin-2, in combination with surgical resection under cardiopulmonary bypass	The patient was well one year after cardiac surgery
**Vikas K Rathi et al. [18]**	7 years	Bradychardia without symptoms	Sinus tachycardia with complete heart block junctional escape rhythm at 42 beats per minute and poor R wave progression in the precordial leads	Increased echogenicity of the ventricular walls, likely due to hypertrophy, with associated myocardial thickening and slightly irregular endocardial margins. No nodular deposits were observed on imaging	NR	The LV demonstrated normal contractility despite marked tumor infiltration of the myocardium, characterized by nodularity throughout the myocardial muscle layers with variable penetration into the endocardium and epicardium. These nodular deposits appeared discrete and ranged from isointense to hyperintense relative to normal myocardium on both T1- and T2-weighted images. Masses in the left and right ventricles, as well as atria, appeared hyperintense on both imaging sequences	NR	NR
**Johann Auer et al. [19]**	9 years	Acute coronary syndrome with sudden onset of chest pain	ST-segment changes, T-wave inversion involving the left precordial leads, and positive cardiac troponin I	A 6 × 3 cm smooth-surfaced, homogeneous echogenic mass involving the anterolateral wall and apex of the LV, accompanied by a small pericardial effusion	Suggestive of metastasis	NR	NR	The patient died six days after admission
**Joseph F. Malouf et al. [20]**	2 years	Asymptomatic	Borderline low voltage and a first-degree atrioventricular block	A globular mass measuring 4.8 × 3.5 cm was identified in the RA, attached broadly to the right atrial free wall	Mass located in the RA, accompanied by nodular densities along the right pleura	NR	Surgical removal of the atrial mass followed by interleukin-2 combined with oral levamisole therapy	NR
**Theodoros D. Karamitsos et al. [21]**	3 years	Dyspnea, fatigue, and peripheral edema	NR	A large mass within the RV, extending from the tricuspid valve to the infundibulum near the pulmonary valve, causing near obliteration of the right ventricular cavity. Right ventricular outflow obstruction was noted, with an estimated systolic pressure of 60 mmHg	NR	Enhancement of the right ventricular mass, suggestive of a highly vascular lesion	Palliative surgery involving debulking of the mass and tricuspid valve repair	The patient died two weeks after surgery
**Antonella Fontana, M.D. [22]**	Unspecified years	Dyspnea and atypical chest pain radiated to the scapulae	Sinus rhythm with new-onset diffuse negative T-waves	A mass attached to the apex of the RV, partially obstructing both the inflow and outflow tracts. The mass appeared as a large, elongated, echogenic lesion measuring 45 × 20 mm, positioned parallel to the interventricular septum. Three-dimensional imaging revealed effective dimensions of 57 × 45 × 40 mm, with intimate attachment to the RV apex and interventricular septum, multiple distal mammillated appendages, and protrusion into the RV outflow tract, causing partial obstruction	Impaired right ventricular filling due to the mass, with widespread dissemination of the primary tumor to distant organs	An expansive endocavitary lesion measuring 60 × 48 × 40 mm, hyperintense on T2-STIR and DP/T1 sequences, occupying the mid-apical third of the right ventricle. The lesion demonstrated moderate, heterogeneous enhancement after administration of paramagnetic contrast, with mammillated appendages extending into and obstructing the right ventricular outflow tract	Experimental treatment protocol including vemurafenib	NR
**Elena Santamarta Liébana et al.** **[23]**	5 years	Asymptomatic	NR	NR	The mass is located in the right atrium	Mass located in the right atrium, extending toward the atrioventricular groove with invasion of the right ventricular wall. No pericardial involvement was observed. The lesion was isointense on T1- and T2-weighted sequences, with moderate gadolinium enhancement	NR	NR
**Gang Cheng et al. [24]**	Brain metastasis at 3 years, with intraventricular spread at 5 years	Supraventricular tachycardia complicated by hemodynamic instability and cardiac arrest	Complete atrioventricular block	Intraventricular thickening observed without evidence of a mass	FDG PET-CT revealed systemic metastases + hypermetabolic focus in the interventricular septum	NR	NR	NR
**Loizos Kontozis et al. [25]**	9 years	Blood pressure of 104/86 mmHg, tachycardia 104 bpm, Jugular venous pressure 10 cm, with prominent a-waves. Presence of an S4, fatigue, malaise, and headaches. Progressively deteriorating exertional dyspnea.	Right atrial enlargement	A large right atrial tumor measuring 5.3 × 4.1 cm, prolapsing through the tricuspid valve and causing leftward displacement of the interventricular septum	No additional pathology was observed	Not reported	Surgically removed	Patient discharged 5 days postoperatively; at 5-month follow-up, transthoracic echocardiography showed no evidence of tumor recurrence
**Aaron J. Gindea et al. [26]**	1 year	Tachycardia at a rate of 110 bpm and tachypnea. Distant heart sounds and a soft systolic murmur were audible across the precordium, and respiratory alkalosis	Sinus tachycardia (110 bpm) with interventricular conduction delay and nonspecific ST–T changes	Both ventricles were filled with an echogenic mass replacing nearly the entire ventricular blood pool, leaving only a small space distal to both atrioventricular valves	No metastases detected	A large mass occupied approximately 70% of both ventricular cavities and extended into the right ventricular outflow tract. T2-weighted images demonstrated a signal intensity difference between the biventricular mass and the surrounding myocardium and interventricular septum	None	The patient died 24 h after the biopsy
**Steven P. Chutelike et al. [27]**	3 years	First heart sound followed by a prominent ejection click in the aortic area, which heralded a grade 316 harsh systolic ejection murmur radiating to the carotids and apex	Normal	A mobile, pedunculated intracavitary mass measuring 3 × 1.5 cm in the LV, protruding into the left ventricular outflow tract and contacting the ventricular septum and anterior mitral valve leaflet during systole	NR	NR	Radiation	Deceased
**MR Carpenter et al. [28]**	17 months	Chest pain and dyspnea	T-wave changes and reduced R-wave amplitude in the anterior chest leads	TEE revealed gross thickening of the interventricular septum (3.2 cm) and a mobile mass within the right ventricle. TOE demonstrated distal septal thickening and two masses in the right ventricle: one adjacent to a papillary muscle, and another frond-like mass located in the ventricular body extending into the outflow tract, causing a 50% reduction in the infundibular diameter	NR	NR	Single fraction radiotherapy to the shoulder, followed by four courses of oral lomustine chemotherapy administered at six-to-eight-week intervals	Clinical remission lasting 11 months, followed by recurrence and death four months later
**James J.H. Chong et al. [29]**	3 years	Exercise-induced hypertension and subjective decrease in exercise capacity	Sinus rhythm with nonspecific anterior T-wave changes	The TEE showed a 6 cm spherical right atrial mass attached to the atrial roof, containing echolucent areas suggestive of necrosis within the mass.	NR	NR	Surgical excision	NR
**Tamer Özülker et al. [30]**	15 days	Asymptomatic	NR	NR	FDG-PET/CT scan revealed intense pathological FDG uptake (SUVmax: 9.8) in the right atrium, corresponding to a solid lesion on axial PET and CT slices.	NR	Surgical excision	NR
**B. Schneider et al. [31]**	7 years	Worsening congestive heart failure requiring immediate mechanical ventilation. Clinical findings included jugular vein distension, soft heart sounds, S3 gallop, dullness and rales at the bases of both lungs, hepatomegaly, ascites, and bilateral leg edema.	Atrial fibrillation and right bundle branch block were noted	Hypertrophied ventricles	NR	NR	NR	NR
**C. Belda-Iniesta et al. [32]**	5 months	Syncope (fainting)	Atrial flutter with a heart rate of 45 bpm	Normal ejection fraction without limitation to left ventricular filling or outflow tract obstruction. Multiple lesions were detected in the myocardium and pericardium. Invasion of the pericardium and myocardium was observed at the confluence of the interatrial and interventricular septa, the typical location of the atrioventricular node	NR	Nodular high-intensity lesion at the confluence of the interatrial and interventricular septa with pericardial infiltration. Metastatic nodular lesion in the atrioventricular nodal region showing gadolinium enhancement.	NR	NR

Abbreviations: Not reported (NR), Transesophageal echocardiography (TEE), Transesophageal Echocardiogram (TOE), Right Atrium (RA), Right Ventricle (RV), Left Ventricle (LV).

## Data Availability

The original contributions presented in this study are included in the article. Further inquiries can be directed to the corresponding author.

## Abbreviations

The following abbreviations are used in this manuscript:

PET-CT	Positron Emission Tomography-Computed Tomography
CMR	Cardiovascular Magnetic Resonance Imaging
LV	Left Ventricular
ECG	Electrocardiogram
LGE	Late Gadolinium Enhancement
CT	Computed Tomography
FDG-PET-CT	Fluorodeoxyglucose-Positron Emission Tomography/Computed Tomography
